# Rearing Water Treatment Induces Microbial Selection Influencing the Microbiota and Pathogen Associated Transcripts of Cod (*Gadus morhua*) Larvae

**DOI:** 10.3389/fmicb.2018.00851

**Published:** 2018-05-01

**Authors:** Ragnhild I. Vestrum, Kari J. K. Attramadal, Per Winge, Keshuai Li, Yngvar Olsen, Atle M. Bones, Olav Vadstein, Ingrid Bakke

**Affiliations:** ^1^Department of Biotechnology and Food Science, Norwegian University of Science and Technology, Trondheim, Norway; ^2^Department of Biology, Norwegian University of Science and Technology, Trondheim, Norway

**Keywords:** microbiota, aquaculture, Atlantic cod, r/K selection, RAS, transcriptomic analysis

## Abstract

We have previously shown that K-selection and microbial stability in the rearing water increases survival and growth of Atlantic cod (*Gadus morhua*) larvae, and that recirculating aquaculture systems (RAS) are compatible with this. Here, we have assessed how water treatment influenced the larval microbiota and host responses at the gene expression level. Cod larvae were reared with two different rearing water systems: a RAS and a flow-through system (FTS). The water microbiota was examined using a 16S rDNA PCR/DGGE strategy. RNA extracted from larvae at 8, 13, and 17 days post hatching was used for microbiota and microarray gene expression analysis. Bacterial cDNA was synthesized and used for 16S rRNA amplicon 454 pyrosequencing of larval microbiota. Both water and larval microbiota differed significantly between the systems, and the larval microbiota appeared to become more dissimilar between systems with time. In total 4 phyla were identified for all larvae: Actinobacteria, Bacteroidetes, Firmicutes, and Proteobacteria. The most profound difference in larval microbiota was a high abundance of *Arcobacter* (Epsilonproteobacteria) in FTS larvae (34 ± 9% of total reads). *Arcobacter* includes several species that are known pathogens for humans and animals. Cod larval transcriptome responses were investigated using an oligonucleotide gene expression microarray covering approximately 24,000 genes. Interestingly, FTS larvae transcriptional profiles revealed an overrepresentation of upregulated transcripts associated with responses to pathogens and infections, such as *c1ql3-like*, *pglyrp-2-like* and zg16, compared to RAS larvae. In conclusion, distinct water treatment systems induced differences in the larval microbiota. FTS larvae showed up-regulation of transcripts associated with responses to microbial stress. These results are consistent with the hypothesis that RAS promotes K-selection and microbial stability by maintaining a microbial load close to the carrying capacity of the system, and ensuring long retention times for both bacteria and water in the system.

## Introduction

Aquaculture of marine fish often face problems with high mortality, infections and malformations/deformities in the production of juveniles. Negative interactions between fish and microbes have been suggested as an important reason for these problems (Vadstein et al., 2013; De Schryver and Vadstein, 2014). Most marine fish larvae in culture, like Atlantic cod (*Gadus morhua*), are immature and fragile upon hatching (Kjørsvik et al., 2004; Magnadóttir et al., 2004; Magnadóttir, 2006), and the larvae are reared in close proximity to high numbers of bacteria and other microorganisms. The fish are challenged not only by specific pathogens, but also by opportunistic bacteria in general (Olafsen, 2001; Vadstein et al., 2003). Toranzo et al. (2005) suggested that most bacterial infections associated with marine fish larvae are caused by opportunistic bacteria that are usually present in the natural environment of the fish. In nature, the environmental conditions are less favorable for the opportunistic bacteria compared to that in an aquaculture system, and these bacteria rarely cause mortalities in natural settings (Toranzo et al., 2005). Due to high loads of organic matter, and uncontrolled bacterial recolonization after disinfection of the rearing water, the microbial community in the rearing water is more unstable, dominated by opportunists, and with higher and more variable bacterial numbers than in nature (Hess-Erga et al., 2010; Attramadal et al., 2014).

To handle these microbial-based problems in marine larval rearing, the focus has been on reducing the number of pathogens in the rearing water. However, the concept of microbial ecology, i.e., understanding the interactions between the microbes and their environment (Konopka, 2009), has normally not been taken into consideration, and the recolonization of the rearing tanks is often ignored. Both feeding and disinfection of the rearing water are part of the normal operation of aquaculture systems, which lead to an increase of organic matter in the rearing tanks and reduced bacterial numbers in the water coming into the fish tanks, respectively. According to the r-/K- selection theory (MacArthur and Wilson, 1967), r-strategists are fast growing opportunists, which thrive in niches with low competition and high levels of nutrients. K-strategists, on the other hand, have a low maximum growth rate, are efficient competitors for nutrients, have high substrate affinity and may create stable communities at biomass levels close to the carrying capacity. Communities established under K-selection are stable to perturbations and with a high diversity compared to communities established under r-selection (Vadstein et al., 1993). A traditional flow through aquaculture system (FTS) will typically be characterized by high and unstable nutrient loads, low hydraulic retention times, and would be expected to select for opportunistic species (r-strategists) (Vadstein et al., 1993; De Schryver and Vadstein, 2014). Removal of the pathogens from the intake water of the system may therefore not have the desired effect on the microbial water quality due to regrowth in the system.

In order to improve the conditions for the fish larvae in the rearing tanks, efforts have been made to stabilize the microbial community in the water after disinfection by applying well-established management strategies and system’s designs based on ecological theory (Skjermo et al., 1997; Attramadal et al., 2014). For example, low disinfection efficiency by moderate ozonation resulted in a more stable microbial community compared to the use of highly efficient UV-irradiation (Attramadal et al., 2012a).

The interest in recirculation aquaculture systems (RAS) has increased during later years (Martins et al., 2010; Badiola et al., 2012; Blancheton et al., 2013; Attramadal et al., 2014). The rationale for this has often been environmental aspects like energy and water reduction, and waste concentration, but the use of such systems has also been proposed as a possible strategy for maintaining microbial control in the production of marine larvae (Attramadal et al., 2012b). In RAS, biofilters contribute to microbial stabilization by maintaining the microbial load close to the carrying capacity. Furthermore, long retention times for water and the bacteria in the system, ensures K-selection of the microbial communitites in the water. K-selection creates a mature microbial community dominated by K-strategists. This is a community which is stable, has high biological control and is able to withstand perturbations below a certain threshold (Vadstein et al., 1993). It has been shown that the considerate use of RAS increases the survival of the fish when compared to traditional FTS (Attramadal et al., 2012b).

Previous studies indicate that the rearing water influences the microbiota of fish larvae (McIntosh et al., 2008; Giatsis et al., 2014, 2015; Bakke et al., 2015). Marine fish drink a substantial amount of water even before the onset of feeding to prevent dehydration. It has been shown that for turbot, the uptake of bacteria is 100 times higher than their drinking rate would imply (Reitan et al., 1998). This indicates that the larvae have an active uptake of microbes. It is well known that the microbiota associated with fish is very important for its health and development (Nayak, 2010). It is therefore likely that different compositions of microbiota will induce different responses in individual fish. Such potential effects can be investigated by transcriptomic analysis of the fish.

Here we examine the effects of FTS and RAS on the microbiota associated with cod larvae. Further, we examine whether the differences in the cod larval microbiota induced distinct host responses at the gene expression level of the fish. We analyzed cod larvae and water samples originating from a first feeding experiment, consisting of two sub-experiments with distinct objectives: (1) Investigate the effect of membrane filtration on bacterial numbers and microbial diversity in a RAS (Wold et al., 2014). (2) Investigate the effect of diet on the gene regulation of cod larvae in an FTS (Li et al., 2015).

## Materials and Methods

The experimental setup for the two original sub-experiments is described below, and details concerning the experiments are given in Wold et al. (2014) and Li et al. (2015).

The study was carried out within the Norwegian animal welfare act guidelines, in accordance with the Animal Welfare Act of December 20th, 1974, amended June 19th, 2009, at a facility with permission to conduct experiments on fish (code 93) provided by the Norwegian Animal Research Authority (NARA). The experiments were approved by NARA.

### Rearing Systems

A first feeding experiment with Atlantic cod larvae was conducted in two different aquaculture systems: one RAS and one FTS. In both systems, sand filtered intake water from the Trondheimsfjord (70 m depth) was used.

**FIGURE 1:**
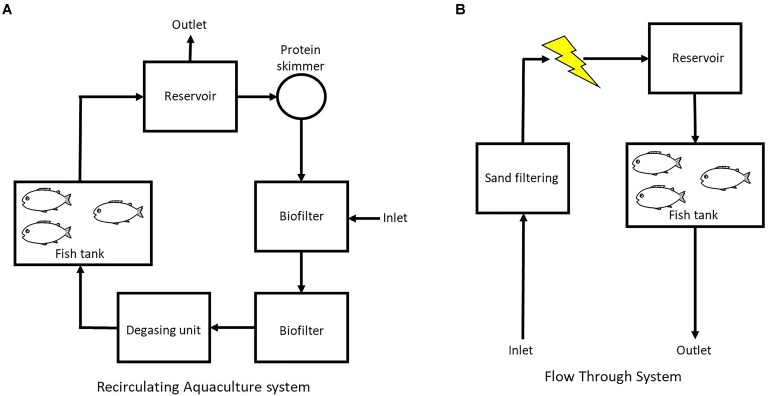
Schematic set up of **(A)** the recirculation aquaculture system (RAS) and **(B)** the flow through system (FTS) used in this study.

In the RAS (**Figure 1A**) the water passed through (flow rate 12.7 L min^-1^) a water reservoir (160 L) and a protein skimmer (80 L, Helgoland 500) before entering two biofilters in series (267 L each) containing biofilm carriers type K1 (AnoxKaldnes^TM^) with a filling fraction of 15% of the biofilter (surface area of 75 m^2^/m^3^_reactor volume_). The water passed through a degassing unit (50 L, vacuum operated) for removal of N_2_ and CO_2_ before entering four replicate rearing tanks (100 L, step wise water volume exchange of 2–3 times d^-1^)(Wold et al., 2014).

In the FTS (**Figure 1B**) the water was treated with UV-irradiation for the inactivation of detrimental bacteria, and kept in a 6 m^3^ aerated reservoir (minimum 12 h hydraulic retention time) with biofilter media (1 m^3^ KMT3, Kaldnes Miljøteknologi AS, Norway) before entering the rearing tanks (100 L, water exchange rate 2–3 × tank volume d^-1^) (Li et al., 2015). In this system two different diets (described below) were used, and there were four tanks for each diet.

### Cod Larvae Rearing

Fertilized Atlantic cod eggs were transported by air from Nofima marine national breeding station, Havbruksstasjonen i Tromsø AS, to NTNU Sealab. The eggs were acclimatized to 6.9°C and disinfected using 400 ppm glutardialdehyde according to Salvesen and Vadstein (1995). After disinfection, the eggs were kept at 7°C in darkness in a 250 L cone bottomed incubator. Two days before hatching, eggs were transferred to 100 L cone bottomed tanks with a density of 100 eggs L^-1^ (temperature 6.5°C). Day 0 was defined as the day when 90% of the embryos hatched [at approximately 90 degree days (dd)].

The cod larvae were kept in darkness until mouth opening at 3 dph, and in continuous light from that point on. The cod larvae were fed rotifers (*Brachionus plicatilis*, Cayman) by a robot system (Storvik Aqua AS) 4 – 6 times per day, to tank densities of 5000–12,000 rotifers L^-1^ from day 2 (FTS) and 3 (RAS) to the end of the experiment. The rotifers were cultivated with Baker’s yeast (*Saccharomyces cerevisiae*) and Rotifer diet (Reed Mariculture), and enriched (long term or short term) with Easy DHA Selco (DSelco, INVE, Aquaculture, Belgium) (Li et al., 2015). The cod larvae in the four replicate RAS tanks were fed long term enriched rotifers. Four FTS tanks (FTS-LT) were fed the same long term enriched as used in the RAS, and the other four (FTS-ST) were fed short term enriched rotifers [detailed description of enrichment protocol in Li et al. (2015)]. *Nannochloropsis oculata* algal paste (Reed Mariculture, 1 mg C L^-1^ final concentration) was added to all tanks at feeding time points (Reitan et al., 1993).

### Sampling

For measurement of size, larvae were collected randomly at 1, 3, 8, 13, and 17 dph. Water and individual larvae for PCR/DGGE analysis were sampled at 5 and 17 dph, and larvae for PCR/454 pyrosequencing were sampled from each tank at 8, 13, and 17 dph. For all these analyses, we chose only to include samples from the RAS and FTS-LT, as those tanks had received the same feed.

Larvae for transcriptomic analysis were sampled from each tank at 8, 13, and 17 dph. For these analyses we chose to include both FTS-LT and FTS-ST as there were no differences in transcript profiles between the two groups (Li et al., 2015).

All larvae were sacrificed by an overdose of Tricaine Methanesulfonate (MS222) before sampling and further processing.

### Larval Size and Survival

Cod larval survival was calculated based on the number of live larvae at the end of each sub-experiment, compared to the number of eggs at the start of the experiment. However, because the two sub-experiments were terminated at different time points, it was not possible to compare the survival in the two systems.

For carbon analysis, larvae were collected randomly including all rearing tanks in each treatment (12 larvae on 1 and 3 dph, and 48 larvae on 8, 13 and 17 dph for each treatment), rinsed in deionized water and transferred into individual pre-weighted tin capsules. The dry weight was calculated based on the carbon analysis by using a CHNSO analyzer (ECS 4010, Costech instruments, Elemental combustion system (series number 260610079). Assuming the carbon content of the larvae dry matter was 43% (Reitan et al., 1993), a conversion factor of 2.34 was applied for conversion of carbon to units of dry matter (Reitan et al., 1993; Wold et al., 2014; Li et al., 2015).

### Analysis and Characterization of Larval and Water Bacterial Communities

#### DNA and RNA Extraction

Cod larvae for DNA extraction were sampled and rinsed in sterile freshwater and thereafter stored individually in Eppendorf tubes at -20°C. Water (50 mL) was sampled and filtered through sterile 0.2 μm hollow fiber syringe filters (DynaGard, Microgon Inc., California) and the filters were stored at -20°C. For RNA extraction individual cod larvae were collected and spotted on a piece of plankton net (100 μm), immediately put into a SafeSeal micro tube (Sarstedt^®^), flash frozen in liquid nitrogen, and stored at -80°C until RNA extraction (Li et al., 2015).

DNA was extracted by use of the DNeasy Blood and Tissue Kit (Qiagen) as described by the manufacturers with minor modifications in the lysis step, as described in Wold et al. (2014), from water samples and individual cod larvae sampled at 5 and 17 dph. Total RNA was extracted from pooled larvae (25 larvae at 8 dph, 17 larvae at 13 dph and 10 larvae at 17 dph) using the RNeasy Mini Kit^®^ from Qiagen, as described in Li et al. (2015).

#### PCR/DGGE

The PCR/DGGE fingerprinting was used as a quick tool to investigate the composition of the microbial communities in the rearing water for three replicate tanks, and larvae samples (one individual per replicate tank at both sampling times) from 5 and 17 dph from the RAS and FTS-LT tanks. Amplified 16S rRNA gene fragments suitable for bacterial DGGE fingerprints of total microbial community DNA samples were obtained using a nested PCR protocol in order to exclude amplification of eukaryotic 18S rDNA (Bakke et al., 2011). The variable region v3 was amplified using the Qiagen Taq PCR core unit kit, and the primers 338F (5′-attaccgcggctgctgg-3′) and 518R (5′-attaccgcggctgctgg-3′) for the internal PCR reaction. PCR products were analyzed by denaturing gradient gel electrophoresis (DGGE), performed with the INGENYphorU DGGE system (Ingeny) using 8% acrylamide gels with a denaturing gradient of 35–55% (where 100% corresponds to 7 M urea and 40% formamide), 0.5 times TAE electrophoresis buffer, at 100 V and 60°C for approximately 18 h (Muyzer et al., 1993). The DGGE gels were stained in Sybr Gold nucleic acid stain (1:10 000 dilution; Molecular Probes) for minimum 1 h, and visualized and photographed in a GenBox geldoc system (Syngene). As a marker for the DGGE gels pooled v3 16S rDNA PCR products from pure cultures were used (*Staphylococcus aureus*, *Ruminococcus obeum, Eubacterium formicigenerans, Ruminococcus productus, Fusobacterium prauznitzii, Clostridium celerescans, Eubacterium plautii, Eubacterium halii*, and *Bifidobacterium longum*). One DGGE gel was run for water samples from three replicate tanks for both rearing systems, in addition to samples from incoming water at both sampling times. A second DGGE gel was run with samples representing larvae from all four replicate tanks from both rearing systems at both sampling points.

#### PCR/454 Pyrosequencing

For more in-depth analysis of the active fraction of the cod larval microbiota, barcoded 454 pyrosequencing was performed based on total RNA extracted from pooled larvae sampled at 8, 13, and 17 dph from the RAS and FTS-LT tanks. cDNA was synthesized by use of Prime Script^TM^ 1st strand cDNA Synthesis Kit (TaKaRa), as described by the manufacturers. Random 6 mers and approximately 1 μg total RNA was used.

Larval samples were prepared for 454 pyrosequencing by amplification of the V4 region of the 16S rRNA gene. To avoid amplification of eukaryotic DNA from cod larvae, a semi nested PCR protocol was used as described by Vik et al. (2013). For both the external (ext.) and internal (int.) amplification the reactions were run for 21 cycles (98°C 15 s, 50°C (ext.)/53°C (int.) 20 s, 72°C 20 s) with 0.6 μM of each primer, 200 μM of each dNTP, 2 mM MgCl_2_, Phusion Hot Start II High-Fidelity DNA Polymerase and reaction buffer from Thermo Scientific. The PCR products were purified and normalized using the SequalPrep^TM^ Normalization Plate Kit. The eluted amplicons were pooled and sequenced on a quarter of a 454 plate with a GS FLX instrument at the Norwegian Sequencing Centre^1^. The resulting pyrosequencing data were deposited at the European Nucleotide Archive (Study accession number PRJEB25934 and sample accession numbers ERS2373973–ERS2373990).

#### Processing and Analysis of DNA Sequence Reads

The sequence data was first processed using the QIIME pipeline version 1.8.0 (Caporaso et al., 2010b) with default parameters. De-noising was performed by flowgram clustering using the program de-noiser and chimeric sequences were identified using UCHIME (Edgar et al., 2011) with default parameters. To retain only reads of sequence lengths of at least 200 bp, minimum quality scores of 25 and no ambiguous bases in the primer sequence, low quality reads were removed in an initial filtering. The sequences were then clustered at 97% similarity, using UCLUST (Edgar, 2010).

A representative sequence from each cluster was used to assign taxonomy by the rdp classifier version 2.3. (Wang et al., 2007) and aligned by PyNAST (Caporaso et al., 2010a) to reference sequences in the Greengenes core set (DeSantis et al., 2006).

### Transcriptomic Analysis: Microarray Design and Hybridization

Microarray design and hybridization was done as described in Li et al. (2015). A custom, Agilent 44 k oligo microarray (A-MEXP-2226, ArrayExpress, EMBL-EBI) (Kleppe et al., 2014) was used. The design of this microarray was in part, based on the Atlantic cod gene set described in Star et al. (2011) and EST sequences from various cod tissues and developmental stages. Two hundred nanograms of total RNA was used to synthesize Cy3 labeled cRNA, using the Low Input Quick Amp Labeling Kit One-Color (Agilent Technologies cat. No. 5190-2305, Santa Clara, CA, United States). cRNA concentration and Cy3 incorporation were measured with a NanoDrop^®^ ND-1000 spectrophotometer. 1.65 μg labeled cRNA was fragmented and hybridized on 4 × 44 k Custom Gene Expression arrays for 18 h at 65°C, using Gene Expression Hybridization Kit (cat. No. 5188–5242) and hybridization oven (G2545A, all from Agilent Technologies). Slides were washed with buffers from Gene Expression Wash Buffer Kit (Agilent cat. No. 5188-5327) and immediately scanned with Agilent scanner (G2505BG25) and data was extracted from the resulting tif images with Feature Extraction software version 4.5.1.

### Statistical Analysis

Denaturing gradient gel electrophoresis gel images were analyzed with the Gel2k software (Norland, 2004) to convert band profiles to histograms, resulting in sample-peak area matrices. To normalize for variations in amounts of PCR product loaded in the wells, individual peak areas for DGGE bands were divided by the sum of the peak areas for the respective DGGE profile. The peak area data was further square root transformed to reduce the impact of strong bands. Statistical analyses were performed using the program package PAST version 2.17 (Hammer et al., 2001). Relative diversity, *J*′ (evenness), Shannon index (*H*′), and band richness (*k*) were calculated and used as indications of the alpha-diversity in the DGGE profiles. Ordination by Principal coordinates (PCoA) based on Bray–Curtis similarity was used to visualize the similarity/dissimilarity in bacterial community profiles among the different samples (beta-diversity). PCoA was also used to visualize similarity/dissimilarity in bacterial community profiles among the different larval samples based on the operational taxonomic units (OTU) table after pyrosequencing.

One-way PERMANOVA and two-way PERMANOVA based on Bray–Curtis similarities were used to test for differences in DGGE and OTU profiles between different groups of samples. Two-way ANOVA was used to test for differences in Shannon’s indices, evenness and OTU richness. SIMPER analysis was performed to identify which OTUs contributed most to dissimilarities between larval samples. The multivariate analyses were performed using the program package PAST version 2.17 (Hammer et al., 2001). The Student’s *t*-test (unpaired) was used to test for differences in weight and size measurements. Differences were considered significant if *p* < 0.05 for all these statistical tests.

The Limma package (version 3.20.1) (Smyth, 2005) and R version 3.0.3 were used for statistical analysis and identification of significant differentially expressed genes. Single color feature expression files from the Agilent microarray scans were imported, and spots identified as feature outliers were excluded from the analysis. Weak or not detected spots were given reduced weight. The median signal intensity of all the spots were log2 transformed and the data were normalized using the quantile method, no background subtraction was performed. A design matrix was created and pair-wise comparisons between the samples, D17R (day 17, RAS) and D17HL (day 17, FTS) was performed. The method of Benjamini and Hochberg (1995) was used to estimate the false discovery rate. Genes with adjusted *p*-value < 0.05 were regarded as significantly differentially expressed. The study is MIAME compliant.

## Results

As described above, the results are based on a first feeding experiment consisting of two sub-experiments (Wold et al., 2014; Li et al., 2015). We investigated the effects that two different water treatment systems, one recirculating aquaculture system (RAS) and one flow through system (FTS), had on water microbiota, larval microbiota, and larval transcript profiles. Water microbiota was investigated using PCR/DGGE, larval microbiota was investigated using PCR/DGGE and 454 pyrosequencing, and the larval gene transcripts were investigated using a custom, Agilent 44 k oligo microarray.

### Growth and Survival of Cod Larvae

Since the two sub-experiments were ended at different time points, comparisons concerning survival were not possible. For the RAS the survival was 12.7 ± 1.6% on 50 dph. The survival for the FTS-LT and FTS-ST was measured to be 46 ± 5% and 43 ± 3%, respectively, on 18 dph.

*t*-tests show that there were no statistical differences in larval size (expressed in terms of increase in larval carbon content (μg C ind^-1^) with time) between the RAS and the FTS-LT on the sampling points (**Table 1**). Only larvae from FTS-LT were used for comparison with RAS, as these groups had received the same kind of feed. Larvae from the FTS-ST were significantly smaller than larvae from the FTS-LT on 8 and 13 dph, however, on 17 dph there were no significant differences.

**Table 1:** Average cod larvae dry weight (μg ind^-1^).

Days post hatching	Dry weight (μg ind^-1^)			Number of larvae	
1	53.2 ± 2.07			12	
	RAS	FTS		RAS	FTS
3	53.2 ± 1.93	50.3 ± 1.45		12	12
	RAS	FTS-LT^∗^	FTS-ST		
8	75.6 ± 11.3	69.1 ± 2.10	56.0 ± 1.63	48	48
13	105.6 ± 3.68	99.9 ± 3.60	86.4 ± 3.68	48	48
17	158.1 ± 7.72	196.7 ± 35.5	155.7 ± 6.39	48	48

*^∗^Only larvae from FTS-LT were used for comparison with RAS. Values represent mean ± SE (standard error).*

### PCR/DGGE Analysis of Larval and Water Bacterial Communities

**FIGURE 2:**
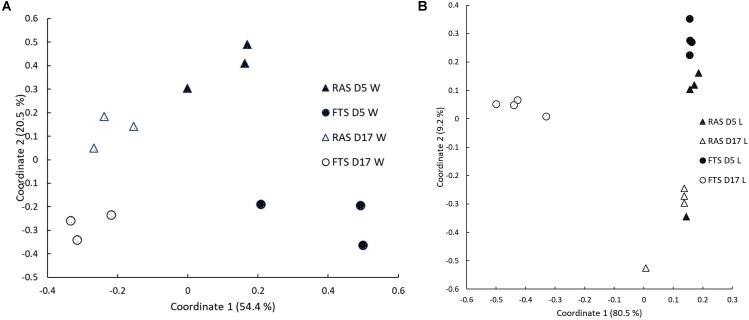
Principal coordinates (PCoA) plot based on Bray–Curtis similarities based on DGGE data for **(A)** water microbiota (W) and **(B)** larval microbiota (L). Both plots show samples taken from RAS and FTS at 5 (D5) and 17 (D17) dph.

The microbial communities in the rearing water and associated with the larvae were investigated by PCR/DGGE analysis for samples from 5 and 17 dph. PCoA plots based on Bray–Curtis similarities (**Figure 2**) indicated that both water and larval microbiota differed between the FTS and the RAS at both 5 and 17 dph. One-way PERMANOVA and two-way PERMANOVA based on Bray–Curtis similarities confirmed that the larval microbiota in the two systems was significantly different at all sampling points (*p* < 0.05) and that the water microbiota differed significantly (*p* = 0.01) between the two systems and that it changed significantly over time (*p* < 0.01). Bray–Curtis similarities for comparisons between RAS and FTS larvae were considerably lower at 17 dph (0.26 ± 0.1) than at 5 dph (0.83 ± 0.14).

### Transcriptomic Analysis of Cod Larvae

The transcriptomics data analyzed in this study were originally generated by Li et al. (2015), who performed transcriptomics analysis of the cod larvae to investigate the effect of lipid content in the feed on larval gene expression. The gene expression data were further analyzed for potential differences between larvae reared in RAS and FTS.

No significant differences in gene expression between RAS and FTS were found on 8 and 13 dph. However, on 17 dph, 20 genes (**Table 2**) showed different expression levels between the two systems. Of these, 19 were significantly up-regulated in the larvae from the FTS compared to the larvae from the RAS, and one uncharacterized gene was down-regulated. Of the genes up-regulated in FTS larvae, 12 are involved in processes coupled to pathogen recognition, infection and immunity responses. In particular, some of the predicted genes are encoding carbohydrate binding proteins or secreted glycosylated proteins with functions related to host-pathogen interactions, such as pglyrp2-like and the zg16-like lectin domain proteins.

**Table 2:** Summary of the significantly (*p* < 0.05) up and down regulated transcripts in cod larvae from the FTS compared to larvae from the the RAS on 17 dph.

Accession number	Gene name	Description	Fold change	Adj. *p*-value
**Involved in pathogen recognition/infection and immunity responses**				
ENSGAUG00000015614_3	*c1ql3-like*	Complement C1q-like protein 3-like	5.34	3.09E-05
ENSGAUG00000005408	*cuzd1-like*	Zona pellucida-like domain-containing protein	4.86	1.94E-02
ENSGAUG00000008126	*zg16*	Zymogen granule membrane protein 16	4.75	1.50E-02
ENSGAUG00000018458_1	*zg16-like*	Zymogen granule membrane protein 16-like	4.69	1.25E-02
ENSGAUG00000014891	*zg16-like*	Zymogen granule membrane protein 16-like	4.48	2.19E-04
ENSGAUG00000005387	*cuzd1-like*	Zona pellucida-like domain-containing protein. Uncharacterized protein	3.09	1.92E-04
ENSGAUG00000012193	*epx*	Eosinophil peroxidase	2.68	3.52E-02
ENSGAUG00000009680	*CTLD-like*	C-type lectin domain protein	1.60	3.89E-03
ENSGAUG00000006359_1	*gimap-like*	GTPase IMAP family protein-like	1.59	1.86E-02
ENSGAUG00000009769	*pglyrp2-like*	*N*-acetylmuramoyl-L-alanine amidase-like	1.48	4.38E-02
ENSGAUG00000014896	*apol1*	ApoL super family protein	1.57	3.67E-02
ENSGAUG00000008563	*ptk6-like*	Protein-tyrosine kinase 6-like	1.64	1.06E-02
**Other**				
ENSGAUG00000003423	*tgm1*	Protein-glutamine gamma-glutamyltransferase K	1.73	3.67E-02
ENSGAUG00000004250	*col6a1-like*	Collagen alpha-1(VI) chain-like	1.64	3.16E-02
ENSGAUG00000003305	*rdh7-like*	Retinol dehydrogenase 7-like	2.12	3.83E-02
ENSGAUG00000016628	*STEAP1*	STEAP family protein	1.39	8.45E-03
ATLCOD1ESTi34197	*tmed1*	Transmembrane emp24 domain-containing protein 1 precursor	1.31	1.93E-02
**Uncharacterized/unknown**				
ENSGAUG00000011018	Unchar.	Alpha-1-acid glycoprotein-like	-1.59	2.26E-02
EX727104.1	Unknown	Unknown transcript	1.72	3.89E-03
EX726615.1	Unknown	Unknown transcript	1.71	1.68E-03

### Characterization of Larval Microbiota by 454 Pyrosequencing

**FIGURE 3:**
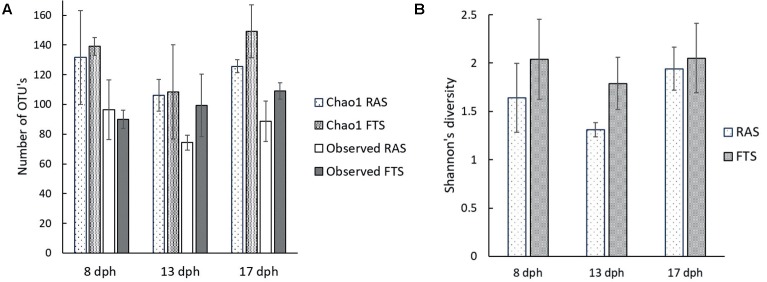
Average richness and diversity indices for cod larval microbiota determined from amplicon pyrosequencing data based on RNA extracts from pooled cod larvae samples: 25 larvae at 8 dph, 17 larvae at 13 dph and 10 larvae at 17 dph in the RAS and FTS-LT. **(A)** Chao1 richness and observed number of OTU’s. **(B)** Shannon’s diversity index.

To investigate possible microbial causes for the upregulation of genes involved in, e.g., pathogen recognition and responses to infection in FTS larvae, we characterized the bacterial communities associated with the cod larvae from both the RAS and the FTS at 8, 13, and 17 dph, in more detail by 454 pyrosequencing of V4 16S rRNA amplicons. After quality trimming and chimera removal, 102056 reads were obtained. Estimated OTU richness (Chao1) and observed number of OTUs (at 97% sequence similarity level) (**Figure 3A**) showed that the sequencing effort across samples covered slightly more than 73% of the estimated richness on average. The difference between the Shannon’s diversity index (**Figure 3B**) for the microbial communities associated with larvae from the FTS compared to larvae from the RAS was between 5 and 30% on average. Two-way ANOVA showed that FTS vs. RAS was the main reason for the variation in Shannon index (*p* = 0.038). However, the average Shannon’s index of all samples decreased by 15.8% from 8 to 13 dph, and increased by 28.4% from 13 to 17 dph. The overall change in the Shannon’s index was by 8.1% increase from 8 to 17 dph (*p* = 0.073).

**FIGURE 4:**
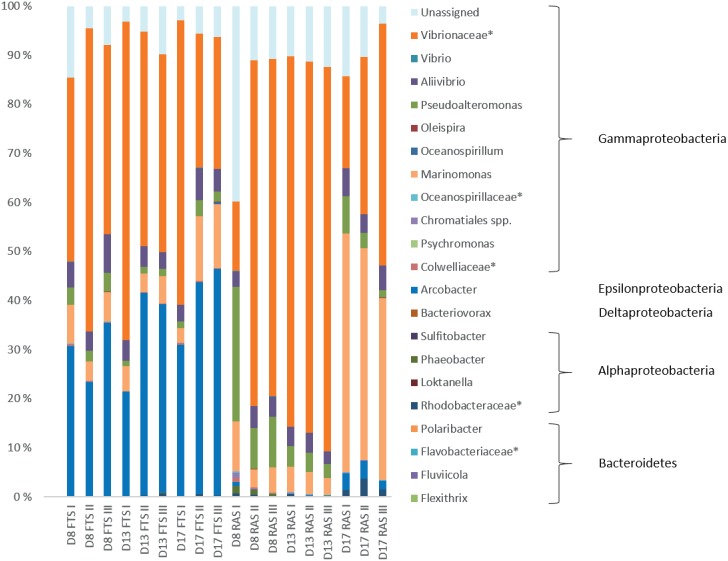
Relative abundances of bacterial genera. Taxa that could not be classified at the genus level are marked by ^∗^. Each sample represents RNA extracts from 25 pooled cod larvae at the age 8 dph, 17 pooled cod larvae at 13 dph and 10 pooled cod larvae at 17 dph (bars labeled D8, D13, and D17, respectively). Bars labeled FTS and RAS represent the flow through system and recirculation aquaculture system, respectively. Only genera represented by a proportion of ≥1% in at least one of the samples are shown.

Cod larval microbiota was compared between the RAS and FTS-LT tanks. In the cod larval microbiota, four bacterial phyla were detected. Bacteroidetes and Proteobacteria were present in all samples. Actinobacteria were present in most larval samples of both systems at all sampling points. However, on the genus level Actinobacteria are missing as all samples contained less than the 1% threshold used. Firmicutes were only found at low abundance (<1%) in one FTS sample. The most abundant class in both systems, at all sampling points, was Gammaproteobacteria (**Figure 4**). In the RAS, this class accounted for on average 84% of the reads, while the other classes were represented by less than 3%. In the FTS, Gammaproteobacteria accounted for on average 58% of the reads. The fraction of Epsilonproteobacteria was much larger in FTS larvae than in the RAS larvae, and accounted for up to 39% of the total reads (17 dph). At the genus level, there was a striking difference between the larval microbiota in the RAS and the FTS samples (**Figure 4**). *Arcobacter* was abundant in the FTS samples (34% on average), and hardly present in the RAS samples (1% on average). On the family level, the Vibrionaceae was most abundant in both systems at almost all sampling points. During the experiment, *Marinomonas* increased in the RAS from between 5 and 10% on 5 and 13 dph, to as much as 48% on 17 dph. OTUs that could not be taxonomically assigned constituted on average 10% of the reads.

SIMPER analysis confirmed the difference between the larval microbiota in the FTS and the RAS. The Epsilonproteobacteria class was represented by 17 different OTUs, all closely related to the genus *Arcobacter.* Two of these OTUs were quite dominating, and they contributed to approximately 30% of the differences between the FTS and the RAS. Looking closer at the taxonomically unassigned reads, we found that they belonged to 64 different OTUs. By using RDP Classifier and BLAST, we found that approximately 25% of the unassigned reads matched OTUs that were closely related to Epsilonproteobacteria, and 19% were closely related to *Arcobacter* specifically. These OTUs were generally more abundant in the microbial communities in the FTS larvae than in the RAS larvae. 76% of the taxonomically unassigned reads were represented by the same OTU.

**FIGURE 5:**
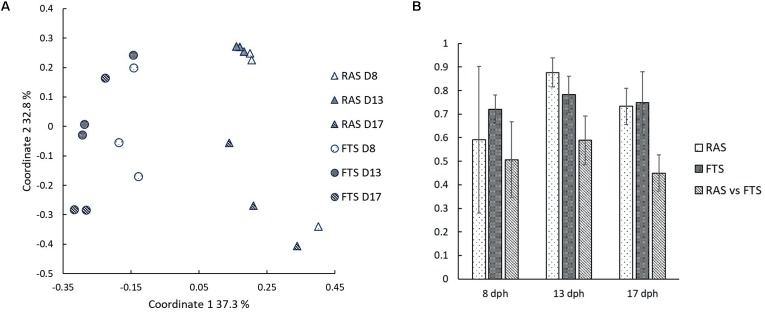
**(A)** Principal coordinates plot based on Bray–Curtis similarities for comparison of larval microbiota in RAS and FTS sampled on 8, 13, and 17 dph. **(B)** Average Bray–Curtis similarities for comparisons of larval microbiota within systems and between systems at 8, 13, and 17 dph. Error bars are standard deviations.

A PCoA plot based on the Bray–Curtis similarity index of pyrosequencing data (**Figure 5**) corroborated the results from the DGGE analysis, and showed that the larval community profiles clustered according to the rearing system, and also according to time point. Bray–Curtis similarities were higher within systems than between, and the tendency was that the similarities within systems increased throughout the experiment, whereas it decreased between systems. Two-way PERMANOVA based on Bray–Curtis similarities confirm that the larval microbiota differed significantly between systems (*p* = 0.0001), and it also changed significantly with time (*p* = 0.0009).

## Discussion

Our hypothesis was that through differences in rearing technology we are able to manipulate the microbiota of the rearing water and that this will affect the microbiota associated with cod larvae. The larval associated microbiota will in turn affect the viability of the larvae. A rearing system dominated by K-selected bacterial communities, in this case RAS, will have a positive effect on the viability of the larvae.

In this study, we investigated the microbiota in the rearing water, microbiota associated with the cod larvae, and larval transcript profiles, as viability variables in a RAS and an FTS. DGGE analyses showed that the microbiota in both the water and associated with the larvae were significantly different in the two systems, and that it changed throughout the experiment. High throughput sequencing of the v3 region of the 16S rRNA gene confirmed this, and revealed that a potentially harmful *Arcobacter* bacterium thrive in the FTS, but not in the RAS. Interestingly, larval transcript profiles showed that certain genes related to pathogen recognition, infection and immune responses were upregulated in larvae from the FTS compared to those from the RAS.

The water treatment differed considerably between the FTS and RAS. For example, RAS included a degassing unit, a protein skimmer, and two moving bed biofilters, whereas the water in FTS was UV treated. All these water treatment units probably affected the water microbiota in the systems. For RAS, the consumption of most of the dissolved organic matter under starvation conditions in the biofilter, the longer hydraulic retention time, and absence of disinfection in the loop probably contributed to K-selection of the water microbiota (Attramadal et al., 2014; Vadstein et al., unpublished), and probably resulted in selection against the *Arcobacter* strain.

In marine fish, the adaptive immune system does not start to mature until 2–3 months after hatching, and during this period the fish rely on the innate immune system. The innate immune system is the first line of defense against pathogens, and is mediated by proteins than recognize specific microbial molecules. These molecules are e.g., lipopolysaccharide (LPS), peptidoglycans, polysaccharides, bacterial DNA, and other molecules that are present on the surface of bacterial cells (Magnadóttir et al., 2004; Magnadóttir, 2006). However, the immune system does not only defend the host against pathogens and tissue damage. It also facilitates colonization by beneficial bacteria in the host, and maintains microbiota-host homeostasis. This is important for the health of the host, and there are many mechanisms working together in order to control the interactions between the host and the microbiota, including the use of peptidoglycan recognition proteins (PGLYRPs or PGRPs) which are able to recognize bacteria via the cell wall component, peptidoglycan (Li et al., 2007; Royet et al., 2011).

Here we found a *N*-acetylmuramoyl-L-alanine amidase-like (*pglyrp-2-like*) transcript, which was upregulated in larvae from the FTS compared to larvae from the RAS. *pglyrp* genes have been identified in several fish species (Royet et al., 2011) and Li et al. (2007) identified four *pglyrp* genes in zebrafish. Through their experiments they showed that the Pglyrps in zebrafish are *N*-acetylmuramoyl-L-alanine amidases that are able to cleave the bond between *N*-acetylmuramic acid and alanine in the peptidoglycan. They are therefore strongly bactericidal for Gram-positive bacteria, including some pathogens, and to some extent bactericidal for Gram-negative bacteria. In our experiment, *pglyrp-2-like* was upregulated in larvae from the FTS, which might indicate that the larvae in the FTS experienced a higher bacterial load or responded more strongly to these bacteria present than larvae in the RAS.

Other proteins that might be important in the host’s response to pathogens are zymogen granule membrane protein 16 (Zg16) and Zg16-like proteins, whose transcripts were upregulated in larvae from the FTS compared to larvae from the RAS. There are at least five *zg16-like* genes in cod, all contain N-terminal leader sequences suggesting that they are targeted for the secretory pathway. Pancreatic acinar cells synthesize, sort, store and secrete a complex mixture of digestive enzymes, which are packed in a condensed and mainly inactive form, into zymogen granules. The content of the granules can then be released by exocytosis triggered by neuronal or hormonal stimulation (Aroso et al., 2015). The ZG16 protein is a 16 kDa soluble protein with a Jacalin-type lectin domain, that was identified in zymogen granules from rat pancreas, and its involvement in the sorting of enzyme proteins to the zymogen granule membrane was demonstrated (Kleene et al., 1999). Lectins are often involved in the recognition and binding of carbohydrates, communication between cells and in host–pathogen interactions. Tateno et al. (2012) detected that the mRNA of human *ZG16* is expressed in the liver, pancreas and small intestine and they demonstrated that the protein binds to mannose, and also to pathogenic fungi coated with mannan. A study by Bergström et al. (2016) showed that ZG16 protein in mice is able to bind to peptidoglycan, the most abundant glycan in bacteria, which triggered aggregation of Gram-positive cells and inhibited mucus penetration by both aggregated and individual cells in the mouse colon. All these studies indicate that ZG16 protein plays an important role in different organisms’ protection against invading non-self cells, which might explain the upregulation of *zg16* in fish from the FTS.

The induction of a transcript encoding a zona pellucida-like domain (ZP-domain) protein (Cuzd1-like) in larvae from FTS was also observed. This is a novel protein with no clear orthologs in mammals, but it is highly conserved within fish and amphibians. The ZP-domain protein contains an unknown N-terminal domain with leader sequence and a C-terminal ZP-domain with homology to human CUB and zona pellucida like domains 1 (CUZD1). ZP-domains are found in many secreted eukaryotic glycoproteins among them the zymogen granule membrane protein GP2 (Jovine et al., 2002). Both CUZD1 and GP2 are expressed in pancreatic tissue in humans and pancreatic autoantibodies targeting GP2 and CUZD1 are used as Crohn’s disease-markers (Pavlidis et al., 2016). Although the function of this protein is unknown, the domain structure suggests that it is a secreted ZP-domain protein with functions probably coupled to host-pathogen interactions.

The transcript that was most upregulated in larvae from the FTS, was complement C1q-like protein 3-like (*c1ql3-like*). This gene codes for a C1q domain protein and it is similar to complement C1q tumor necrosis factor-related protein 3-like earlier found in Nile tilapia (*Oreochromis niloticus*) (NCBI Reference Sequence: XP_003458730.2). The complement system is an important part of the innate immune system, consisting of an array of proteins, and well known for its ability to kill pathogens (Holland and Lambris, 2002). C1q is part of the C1 protein complex, which is the first complement component. Complement activation can take place in three different ways, and the classical complement activations is initiated when IgM or IgG bind to C1q. This is the first step in a cascade of reactions which results in three possible outcomes: (1) C3b serves as an opsonin which increases phagocytosis; (2) C5a contributes to the inflammation process by attracting phagocytic cells to the cite of infection; and (3) the membrane attack complex (MAC), which is able to create pores in the cell membrane, leading to cell lysis and death, self assembles (Holland and Lambris, 2002).

The fact that transcripts related to pathogen recognition and immune responses were upregulated in the FTS larvae, might suggest that the larvae in this system had to endure a higher amount of potentially harmful bacteria, than the larvae in the RAS. Based on growth and visual observations, the cod larvae in our study were healthy and in good shape in both rearing systems. However, for the larvae in the FTS immune responses are induced. This inspired us to investigate possible underlying reasons – were there differences in the microbiota profiles in the two systems that might explain these observations?

It has long been known that several *Vibrio* species can cause diseases in marine species, and vibriosis caused by *Vibrio anguillarum* is the most common one (Colwell and Grimes, 1984). In both the RAS and the FTS Gammaproteobacteria was the most abundant class, and the Vibrionaceae family was dominating, but this did not seem to affect the cod larvae negatively. The main difference between the FTS and the RAS was the high abundance of *Arcobacter* in the FTS larvae. In the RAS larvae this genus was barely present and it is therefore reasonable to assume that it might be responsible for the host responses discussed above.

*Arcobacter* belongs to the Epsilonproteobacteria class, which typically includes strains that inhabit the digestive tract of animals and humans and serve as symbionts and pathogens and several strains of the genus have been recognized as enteropathogens (Collado and Figueras, 2011). Most clinical cases where animals have been affected are restricted to mammals (Collado and Figueras, 2011). However, one study shows that a strain of *Arcobacter* caused death in rainbow trout (Yildiz and Aydin, 2006). Because our experiment was ended at 18 dph, it is difficult to predict what would have happened at a later stage, but our data indicated that the fraction of *Arcobacter* in the FTS increased with time. Such a development might potentially cause detrimental conditions for the cod larvae at a later stage. In line with this observation, Califano et al. (2017) found that OTUs representing potential pathogenic *Pseudomonas* and *Streptococcus* species, were more abundant at 34 than 2 dph.

*Arcobacter* has been identified as an abundant genus in cod larval microbiota in previous experiments using FTS (Bakke et al., 2015; Forberg et al., 2016). Bakke et al. (2015) studied the microbiota associated with cod larvae at different developmental stages, and they found that an *Arcobacter* OTU represented between 17 and 77% of the total reads for cod larvae at 17 and 32dph. Forberg et al. (2016) also found *Arcobacter* to be the dominating genus when investigating the microbiota associated with small and large cod larvae. In both these studies, the authors speculated that the *Arcobacter* was a part of the commensal microbiota of cod larvae. In this study, however, we found that the colonization of the cod larvae by *Arcobacter* was promoted in the FTS, and that this colonization probably induced specific immune responses of the larvae in the *Arcobacter-*colonized system.

We suggest that *Arcobacter* represents opportunistic bacteria that are selected for in FTS. A water treatment system favoring opportunistic bacteria might also select for opportunistic bacteria able to colonize the fish, which might become a problem for the fish. There were no significant differences in growth between RAS larvae and FTS larvae on 17 dph, but this does not rule out the possibility that the negative consequences of the *Arcobacter* colonization might appear at a later stage. Experiments have shown that cod larvae reared in FTS have lower survival than cod larvae reared in RAS (Attramadal et al., 2012b), and based on our results we believe that this is due to the blooming of opportunistic bacteria like *Arcobacter* in FTS.

The findings in this study indicate that systems design and optimization of microbial water quality is particularly important in the early phase of marine juvenile production. However, microbial water quality is not simply a question about RAS or FTS, as the performance of any rearing facility will depend on system configuration and operation. Also in FTS K-selection can be secured by the microbial maturation technique (Salvesen and Vadstein, 1995; Skjermo et al., 1997; Attramadal et al., 2014). Further, it is possible to improve microbial stability by increasing the carrying capacity in a microbially matured FTS. In RAS it is possible to compromise the K-selection by introduction of disinfection just before the rearing tanks (Attramadal et al., 2012a; Vadstein et al., unpublished). Such operational variables influences the microbiota of the water and affect the viability of the larvae (reviewed in Vadstein et al., unpublished). Thus, both FTS and RAS can be run in with a system configuration and operation practice which promote K-selection and thus benign fish-microbiota interactions (Vadstein et al., unpublished). In the present study, the consumption of most of the dissolved organic matter under starvation conditions in the biofilter, the longer hydraulic retention time, and absence of disinfection in the loop of the RAS system probably contributed to K-selection of the water microbiota (Attramadal et al., 2014; Vadstein et al., unpublished), and selection against the *Arcobacter* strain, whereas in FTS, r-selection took place due to uncontrolled recolonization of the intake water after disinfection.

## Author Contributions

RV performed the analyses of DGGE gels, cDNA synthesis, created library for 454 pyrosequencing, analyses of DNA sequence reads, statistical analyses, and wrote the initial version of the manuscript. KA contributed to conception and design of the study, first feeding experiments, sampling, and discussion of results. PW contributed to transcriptomic analysis, microarray design and hybridization, statistical analyses of microarray results, and wrote the sections of the manuscript. KL carried out first feeding experiments, sampling, size and survival calculations, and DNA and RNA extraction. YO and AB contributed to conception and design of the study. OV contributed to conception and design of the study and statistical analyses. IB contributed to conception and design of the study and performed DGGE and processing of DNA sequence reads after 454 pyrosequencing. All authors contributed to manuscript revision, read, and approved the submitted version.

## Conflict of Interest Statement

The authors declare that the research was conducted in the absence of any commercial or financial relationships that could be construed as a potential conflict of interest.

## Footnotes

1 http://www.sequencing.uio.no
